# Stromal cells in normal colon and colon cancers: importance of thyroid hormone signaling

**DOI:** 10.1038/s41419-025-08005-0

**Published:** 2025-10-06

**Authors:** Mathieu Reslinger, Michelina Plateroti

**Affiliations:** Institute of Genetics and Molecular and Cellular Biology, CNRS UMR7104—INSERM U1258—Université de Strasbourg, Illkirch, France

**Keywords:** Cancer microenvironment, Cell signalling

## Abstract

Thyroid hormones (THs, namely T3 and T4) regulate intestinal development and homeostasis *via* thyroid hormone nuclear receptors (TRs), which are T3-modulated transcription factors. Previous work has highlighted the importance of THs and the TRα1 receptor in intestinal stem cell biology and tumor formation, through actions on WNT, NOTCH, and BMP signaling pathways, which mediate epithelial-stromal cell interactions. Recent findings underscore the critical role of stromal cells in maintaining homeostasis and interacting with colonic stem cells. Stromal cells, especially cancer-associated fibroblasts (CAFs), are also essential in colorectal cancer (CRC). While the TH/TR signaling on gut epithelia-stromal interactions is well characterized in amphibians during the TH-dependent metamorphosis process, its function in the normal mammalian colon is still poorly defined, and in CRCs, it remains underexplored. In addition, it is worth underlining that TRα1 mutations in patients are responsible for Resistance to Thyroid Hormone-α (RTH-α) syndrome. This syndrome is a complex pathology that recapitulates typical traits of hypothyroidism, including gut malfunction. Up to now, very little is known about the cellular alterations in the gut of RTH-α patients. This review summarizes recent studies on the roles of T3 and TRα1 in colon physiopathology, with an emphasis on epithelial/stromal or tumor/stromal interactions *via* cell-cell signaling.

## Facts

- The TRα1 nuclear receptor is the principal mediator of the thyroid hormone actions in the small intestinal and colonic mucosae.
- TRα1 is expressed in colon epithelial and stromal cells and is increased in human colorectal cancer, both in tumor and stromal cells.
- During metamorphosis, thyroid hormone-dependent epithelial/stromal interactions are fundamental for intestinal maturation in amphibians.

## Open questions

- Signal exchanges mediated by thyroid hormones and TRα1 in mammalian gut pathophysiology are poorly studied.
- The relevance of thyroid hormone-mediated tumor/stromal interactions in human colorectal cancer has never been analyzed.
- Targeting TRα1 action by blocking tumor/stromal interactions in colorectal cancer may represent a therapeutic strategy.

## Introduction

### The functional organization of the intestinal mucosa

**Fig. 1:**
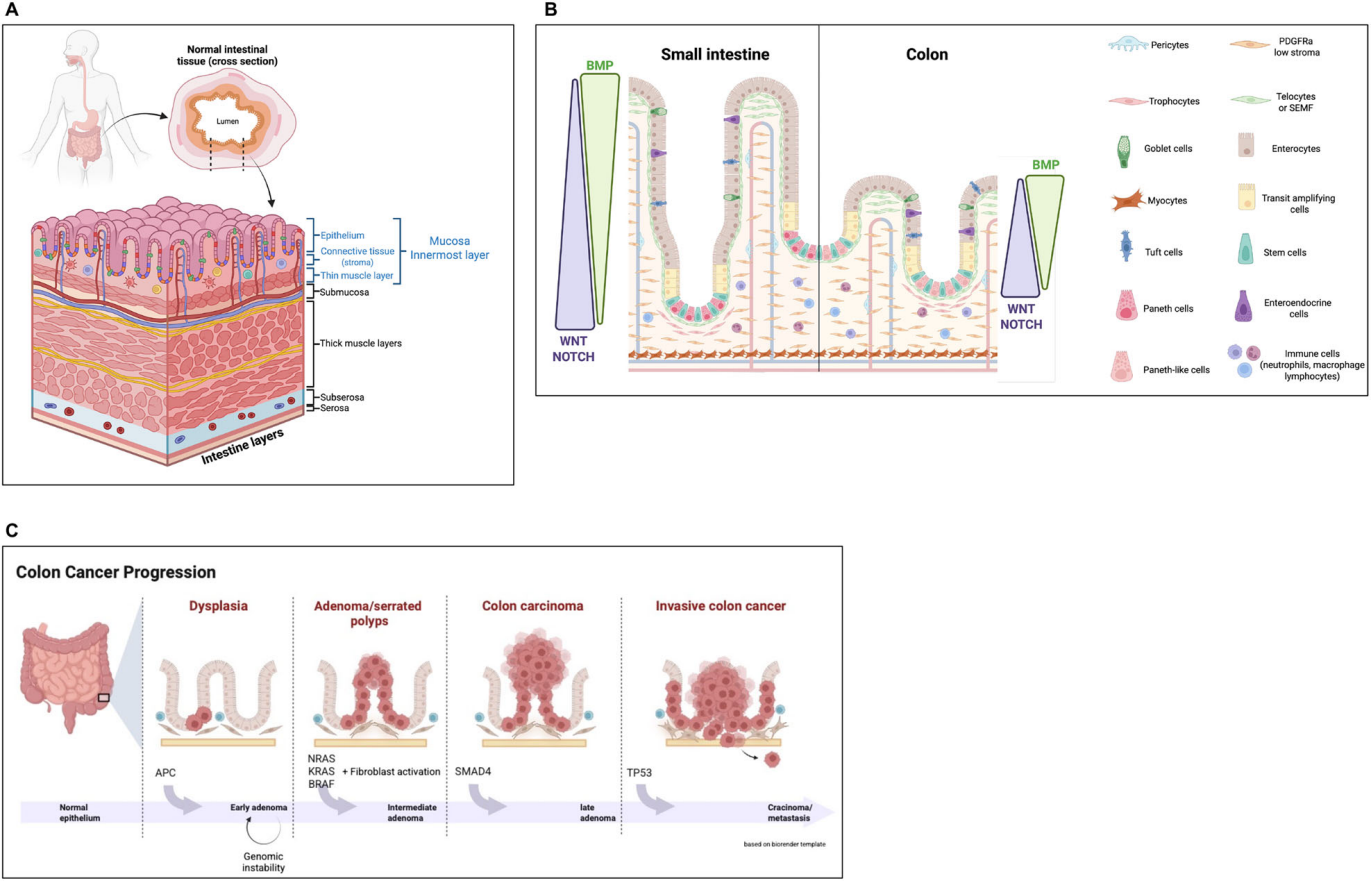
Organization of the intestinal mucosa and the tumor initiation and progression process. **A** Structural organization of the intestinal wall with the five principal layers from lumen to serosa. **B** Schematic representation of the small intestine and colonic epithelium. The spatial organization of the epithelium is regulated by three principal signaling pathways: NOTCH and WNT, which are active at the base of the crypts, and BMP, which is active close to the luminal surface. **C** Tumor initiation and progression from normal epithelium to invasive carcinoma. Starting from different stages of dysplasia, adenoma/serrated polyps, carcinoma, and invasive cancer, the model of colorectal cancer progression involves multiple genetic and cellular changes. The key genetic alterations involved are mutations in *APC*, *KRAS, NRAS, BRAF*, *SMAD4*, and *TP53*. Fibroblast activation supports this process. Created in BioRender (Plateroti, M., 2025; https://BioRender.com/u0w55s6).

In mammals, the intestinal mucosa comprises multiple tissues, including the external muscle layers, the enteric nervous system, the connective tissue and the epithelial tissue [1] (Fig. 1A). The epithelium of the small intestine and colon performs important functions in food digestion and nutrient absorption, and acts as a barrier against pathogenic bacteria and toxins present in the intestinal lumen. The epithelial cells are continuously renewed by proliferating progenitors derived from multipotent intestinal stem cells (ISCs), with a turnover time of 3–5 days under normal conditions [1]. This tissue is organized into two morphologically and functionally distinct structures: the differentiated compartment composed of finger-like villi that protrude into the lumen (small intestine) or flat surface (colon), and the proliferative compartment formed by the crypts (small intestine) or the bottom of the crypts (colon), where reside the progenitors and the ISCs (Fig. 1B). As said, the intestinal mucosa is composed of various tissues in addition to the epithelial layer. In this review, we focus on the different types of mesenchymal-derived fibroblasts. Considered for a long time only structural supportive elements, in both the differentiated and the stem compartments, these cells actively participate in maintaining intestinal epithelial homeostasis by providing specific growth factors and chemokines [2, 3]. In addition, recent advances have revealed a great diversity of fibroblast cell types characterized by the expression of specific markers, which are fundamental in maintaining the ISC and their activity [2, 4, 5]. Therefore, they are now considered essential elements of the ISC niche by contributing to epithelial-mesenchymal instructive signaling [6].

In addition to their function in the normal intestine, the fibroblasts are also essential for intestinal tumor biology [6]. Multiple cell types constituting the supportive network known as the tumor microenvironment (TME) are recruited during cancer initiation and progression to perform essential pro-tumorigenic functions. These cells allow the newly transformed cancer cells to evade immune system recognition and create an environment in which tumors can grow uncontrolled [6]. Fibroblasts are among the first cell types to be recruited within the TME and start the crosstalk with cancer cells upon activation. Activated fibroblasts, also known as cancer-associated fibroblasts (CAFs), can promote cancer progression and immune evasion through the secretion of immunomodulatory molecules, physical interactions with immune cells, or remodeling of the extracellular matrix (ECM) [6]. Like normal intestine, CAFs include multiple cell populations with specific and sometimes opposite pro-tumoral or anti-tumoral functions [6, 7]. The high heterogeneity and plasticity of CAFs can explain these contradictory roles. Indeed, depending on their origin, cancer type, and tumor stage, distinct CAF subtypes have been identified as myofibroblast-like, pro-inflammatory, or antigen-presenting, which perform different functions [7, 8]. These diverse properties are important for shaping the intra-tumoral architecture and contributing to functional changes in tumor cell behavior. It now appears to be of utmost importance to better define the properties of these different cell types and how they change and co-evolve with epithelial and cancer cells during intestinal tumor development and progression.

The process of intestinal tumorigenesis depends on the acquisition of sporadic mutations in key genes and their accumulation over time, as well as on genomic alterations and rearrangements [8]. Several genes involved in normal epithelium-mesenchymal signal exchanges, important for intestinal homeostasis, are mutated and belong to the Wingless and Int-1 (WNT)/β-catenin, Epidermal Growth Factor (EGF), Bone Morphogenetic Protein (BMP), and Transforming Growth Factor beta (TGFβ) signaling pathways [6, 9] (Fig. 1C). Thus, a crucial step in tumor formation is the loss of this balanced signal exchange. In particular, the constitutive activation of WNT/β-catenin and EGF or the impairment of BMP and TGFβ favors the maintenance of undifferentiated epithelial cells, promoting abnormal cell proliferation, one of the first steps leading to cell transformation [1, 2]. Interestingly, some environmental factors, such as hormones, can participate in establishing and maintaining this equilibrium, and their alterations are involved in intestinal tumor biology [10–12]. This is particularly the case of the thyroid hormones (THs) and their nuclear receptors TRs [11, 13], which act as T3-modulated transcription factors [14]. Research conducted in various labs, including our, has demonstrated that the TRα1 receptor plays a crucial role in regulating intestinal development and homeostasis, and its upregulation is associated with tumor development. In contrast, its loss of function has the opposite effect [15, 16]. Among the mechanisms involved, TRα1 controls the biology of the ISCs and of epithelial progenitors and mesenchymal-derived cells, including fibroblasts and smooth muscle cells [17–19]. This is achieved by modulation of several signaling pathways, including the WNT, BMP, and Hedgehog (HH), that involve both epithelial and mesenchymal cells [17–19]. In amphibian metamorphosis, the paradigm illustrating the function of the hormone T3 during development, the steps of signal exchange between the different intestinal tissues and the outcome are quite well described [20]. On the contrary, in mouse models it still needs to be better analyzed, especially in the context of intestinal cancers. This review aims to critically summarize the current knowledge of the different fibroblast cell types and the impact of the hormone T3 and its nuclear receptor TRα1 in normal intestine and cancer, with a special focus on the epithelial/tumor-mesenchymal signaling.

### The thyroid hormones and their nuclear receptors TRs

This section summarizes the key elements of the TH-dependent signaling through the TRs. For recent and detailed reviews, see [21, 22].

**Fig. 2:**
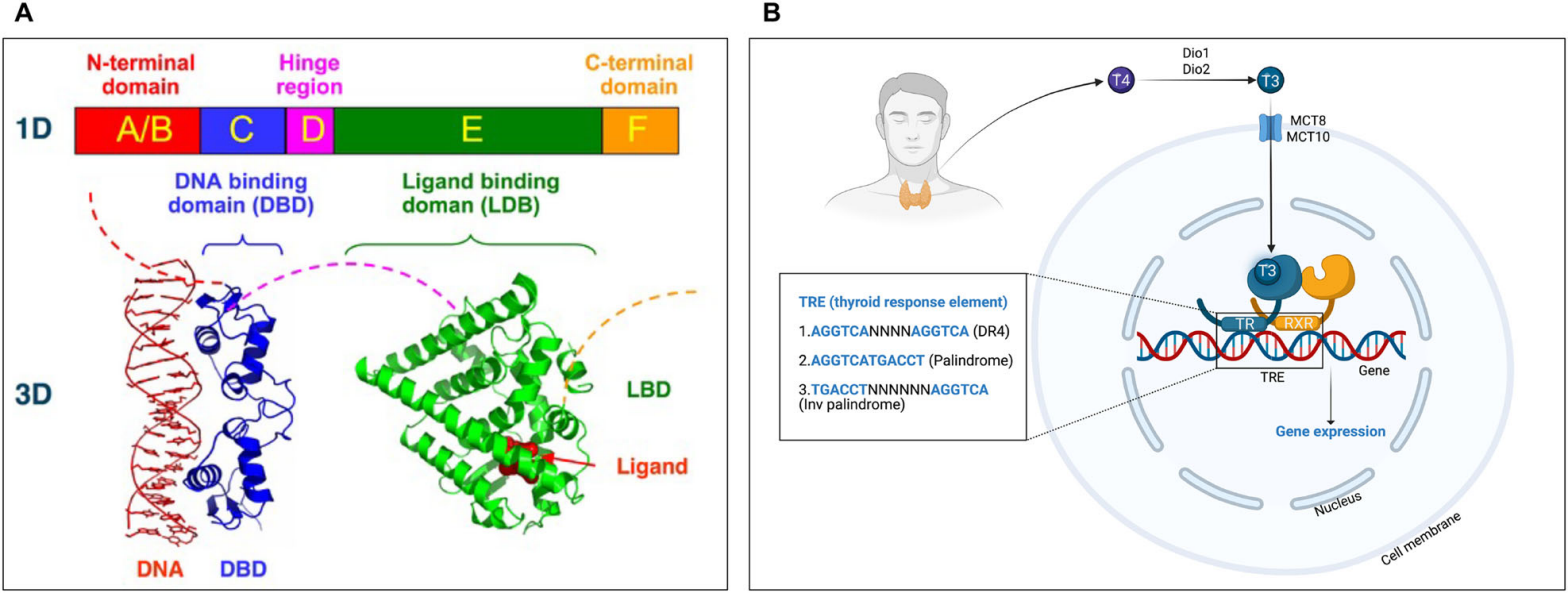
Structural and functional organization of the TRs. **A** Modular organization of the TRs (upper part) and 3D organization (lower part) of the DNA-binding domain (DBD) and the ligand-binding domain (LBD). **B** Molecular mechanism of TR action on target genes. The hormone T4, secreted by the thyroid gland, is converted into the active T3 hormone by the deiodinases DIO1 or DIO2. T3 is transported into target cells *via* specific transporters, such as MCT8 and MCT10, and then binds to TRs in the nucleus. To modulate transcription, TRs form heterodimers with RXR nuclear receptors and bind to thyroid hormone-responsive elements (TREs), which consist of two half-site repeats arranged in a specific orientation. Created in BioRender (Plateroti, M., 2025; https://BioRender.com/8enc5lf).

The thyroid gland produces the T3 and T4 hormones and their circulating concentration are finely controlled by the hypothalamic–pituitary–thyroid axis [23]. Several functional steps are important for the physiology of THs, including (i) THs secretion into the blood and their transport [24], (ii) their uptake into the cells mediated by specific plasma-membrane transporters [25], (iii) T3 and T4 activation or inactivation by the deiodinase selenoenzymes within the cells [26, 27] and (iv) T3-high affinity binding to TRs in cell nuclei to modulate transcription of target genes [28, 29]. TRs control gene transcription through direct DNA binding, now considered the canonical type 1 regulation [30]. Indeed, additional TR-mediated gene regulations exist, including participation in protein complexes independent of DNA binding or of recruitment to DNA [30]. We concentrate mainly on TR-dependent type 1 regulation in this review. From a structural perspective, the TRs have a modular organization, typical of nuclear hormone receptor transcription factors [29] (Fig. 2A). The N-terminal A/B domain permits cofactors binding and contains a T3-independent activation function 1 (AF1) region. The C domain is composed of two zinc finger structures that bind specific DNA elements within target genes, while the D domain, also known as the hinge region, links the C and E domains and harbors a nuclear localization signal. The E domain contains the ligand- and co-factor-binding regions, as well as the T3-dependent activation function 2 (AF-2), which determines the downstream transcriptional response (gene activation or repression) depending on the presence or absence of T3 and on the recruitment of co-activators or co-repressors [28]. To regulate the expression of target genes, TRs bind to genomic regulatory regions called thyroid hormone response elements (TREs), which consist of repetitions of hexameric nucleotide half-sites organized as direct repeats separated by four nucleotides (DR4) or as direct or inverted palindromes [28] (Fig. 2B).

TRs are highly conserved transcription factors expressed in numerous tissues in several species, including mammals, amphibians, fish, and birds [31]. In mammals, the *THRA/Thra* and *THRB/Thrb* genes encode TRα and TRβ protein isoforms, respectively, which are generated through alternative splicing. The *THRA/Thra* locus encodes the TRα1 protein, a true T3 nuclear receptor that possesses DNA- and ligand-binding domains, and the TRα2 isoform that only displays the DNA-binding domain but lacks the ligand-binding domain [31]. While the function of TRα2 remains poorly defined [32], an essential role for TRα1 has been documented during development [31, 33, 34] and in specific adult organs, such as the heart, brain, and intestine [31]. In addition to TRα1 and TRα2, the *THRA/Thra* locus generates two truncated protein isoforms, TRΔα1 and TRΔα2, from an internal promoter. They lack both the DNA- and ligand-binding domains [35]. Together with TRα2, these truncated TRΔ isoforms may block the functionality of the TR receptors, and their role was described in the murine intestine [32, 36]. The *THRB/Thrb* gene encodes TRβ1 and TRβ2 T3 nuclear receptors [31]. TRβ1 and/or TRβ2 play a significant role in metabolic functions (liver), sensorial functions (retina, developing inner ear), and at the level of the hypothalamic-pituitary axis (i.e., TRH neurons and the pituitary gland) [31, 37].

## Heterogeneity of fibroblast populations in normal colon and cancer

**Fig. 3:**
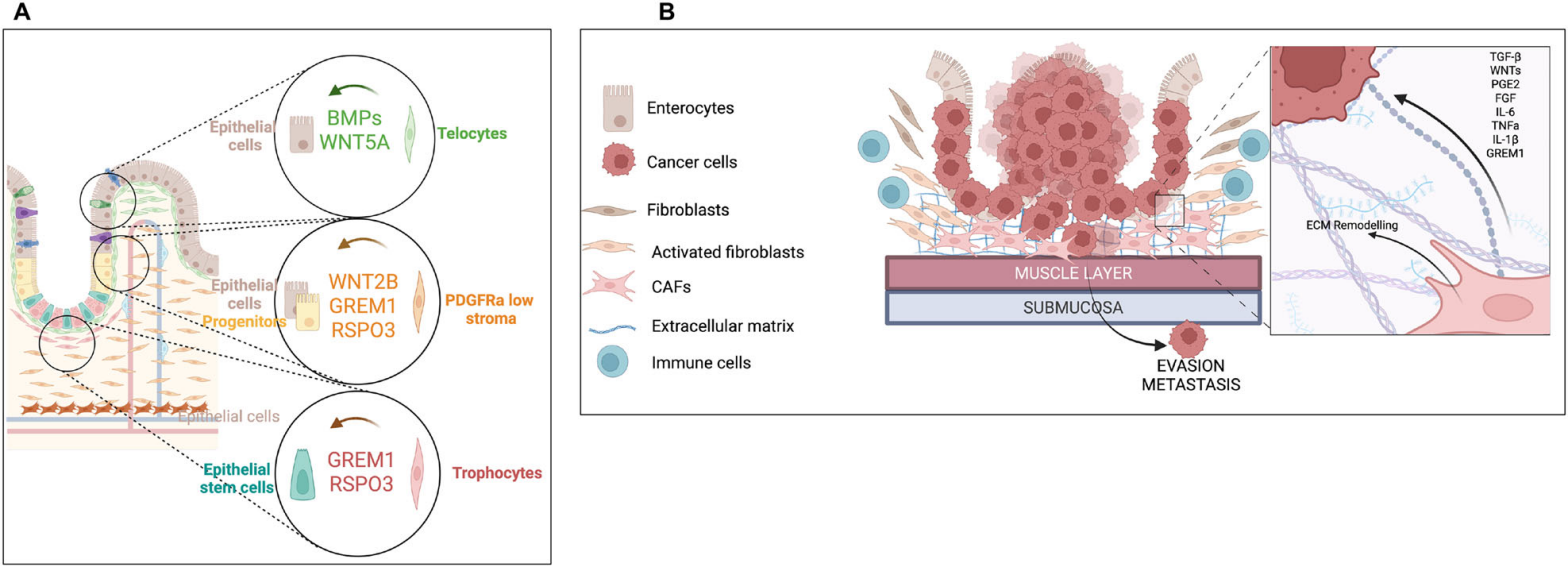
Highlights of the epithelial-stromal interactions in normal colon and colon cancer. **A** The different colonic stromal fibroblasts regulate the epithelial biology along the vertical axis. This control helps maintain epithelial homeostasis through secreted factors and ECM organization. **B** Schematic representation of CAFs in CRCs and their signaling to tumor cells through secreted molecules and ECM remodeling. Created in BioRender (Plateroti, M., 2025; https://BioRender.com/z2xggoj.

The colon mucosa is composed of 3 main compartments: the epithelium, a tissue monolayer composed of different cell types, the connective tissue (or *lamina propria*) that contains the stroma, and a thin muscle layer called muscularis mucosae [2, 3] (Figs. 1A, 3A). In this review, we are focusing more specifically on the *lamina propria*.

The colon stroma supports the epithelium mechanically, provides immunity, and sustains a metabolic network. Fibroblasts constitute an important population within the stroma, particularly those located around the base of the crypts. In fact, they are crucial for regulating crypt cells by secreting factors like WNT ligands, R-spondins (RSPOs), Noggin, and BMP [2], which govern stem cell behavior and differentiation (Fig. 3A). They also contribute to ECM production, forming a network of glycoproteins and proteoglycans that provide structural support, mediate cell adhesion *via* integrins, and facilitate cell signaling and communication [38–40]. Maintaining the integrity of the ISC niche is essential for preserving crypt homeostasis and preventing tumor formation [39, 40].

### The major intestinal stromal cell populations

Five main populations of stromal cells have been identified in the colon, playing both structural and functional roles in maintaining colon crypt epithelial biology, including ISC activity, proliferation, and differentiation (Fig. 3**;** details also in Table 1). The definition of distinct fibroblast subsets in the intestinal mucosa has been difficult due to overlapping markers. However, single-cell transcriptomics has helped to identify fibroblast subtypes in the healthy human colon [39]. These subsets include myofibroblasts and other populations, which have distinct gene expression profiles related to the ECM, basement membrane, and immune processes [39, 41]. These findings clearly highlight the diversity of fibroblasts and their specific functions. Below a summary of some features of three key fibroblast populations: the telocytes, the Platelet-Derived Growth Factor receptor alpha (PDGFRα)-low fibroblasts, and the trophocytes. All these fibroblast populations crosstalk with the epithelium and are important for colon homeostasis. Moreover, we briefly describe additional mesenchymal-derived cells, such as myocytes, pericytes, and endothelial cells, that also contribute to colon physiology.

**Table 1:** Features and localization of the different colon stromal cell populations.

Name of cell type	Localization	Role	Principal Markers
Telocytes	Apex of mucosa	Support, signals to epithelium, epithelium differentiation	PDGFRα-high, GLI1, FOXL1, PDPN
PDGFRα-low cells	Apex, middle and bottom of the mucosa	Support, signals to epithelium	PDGFRα-low, GLI1, CD34, PDPN
Trophocytes	Bottom of the mucosa	Support, signals to epithelium, stem cell activity	PDGFRα-low, CD81, CD34, GREM1, GLI1, PDPN
Myocytes	Smooth muscle layers	Gut motility	ACTA2, GLI1, MYH11, DES
Pericytes/endothelial cells	Blood vessels	Angiogenesis, vessel integrity	PECAM, LYVE1, RSG4

Telocytes, also known as sub-epithelial myofibroblasts (SEMF), are in direct contact with the epithelium beneath the epithelial barrier (Fig. 3A). They have ten- to hundred micrometer-long cytoplasmic extensions called “telopodes” [42, 43] and form a layer from the crypt base to the top, with higher densities at the crypt’s apex. Originally thought to have a purely structural role in maintaining the epithelial tissue, telocytes are now recognized for their signaling functions. They secrete BMPs at the top of the colonic crypt, helping maintain epithelial cell differentiation. These cells express high levels of PDGFRα along with markers like Vimentin (VIM), Glioma-Associated Oncogene Homolog 1 (GLI1), Forkhead Box L1 (FOXL1), and Podoplanin (PDPN) [2]. FOXL1-positive telocytes have extensive cytoplasm and lengthy processes spanning the vertical epithelial axis. When FOXL1-positive cells are ablated in a mouse model, the intestinal architecture is significantly altered, resulting in reduced crypt and villus length, as well as depletion of stem and proliferative cells. Blocking WNT secretion in FOXL1-positive cells has similar effects, indicating the functional link between these two events [4]. Single-cell RNA sequencing (scRNA-seq) has revealed that telocytes express varying molecular signatures along the vertical epithelial axis, aggregating at the top with high PDGFRα expression and secreting BMPs and noncanonical WNT5A ligand [2, 4, 44].

PDGFRα low fibroblasts are a highly plastic population located in the middle portion of the colon crypts (Fig. 3A) and represent the most heterogeneous fibroblast group [2, 45]. Their role is primarily supportive, though emerging evidence suggests they also participate in signaling processes, a function that is beginning to be understood. Depending on their location, they create a gradient of signaling pathways, including WNT and BMP [45]. Markers including VIM, GLI1, low PDGFRα, CD34, and PDPN identify these fibroblasts. PDGFRα low fibroblasts residing in the lamina propria and near the muscularis externa, express WNT2B, Gremlin 1 (GREM1), and RSPO3 and have been shown to support small intestinal organoids in mouse models. However, BMP exposure inhibits this function by reducing the expression of GREM1 and RSPO3. sc-RNA-seq studies have revealed similar stromal clusters in human intestinal mesenchymal populations, with regional variations along the gut [2, 44, 46].

Trophocytes are located at the bottom of colonic crypts, near ISCs and the *lamina propria* (Fig. 3A). Unlike PDGFRα-low fibroblasts, trophocytes are a rare cell population characterized by their proximity to the ISCs and the co-expression of CD81, CD34, Atypical Chemokine Receptor 4 (ACKR4), and GREM1 markers [2, 45, 47]. Trophocytes are crucial for maintaining the colon epithelium by sending inhibitory BMP signals to the epithelial cells at the crypt base, which helps regulate ISC activity [4, 45, 48]. It is also important to underly their function in supporting and facilitating epithelium repair after an injury [4, 40, 44, 46, 48].

Myocytes, which form the smooth muscle tissue of the muscularis mucosae, are located beneath the trophocytes and play a critical role in gut motility and peristalsis. These cells are organized within smooth muscle fibers along blood vessels and lymphatic vessels, extending into the submucosa. They express markers such as Actin Alpha 2 Smooth Muscle (ACTA2), VIM, GLI1, Myosin Heavy Chain 11 (MYH11), and Desmin (DES) [44, 46].

Finally, pericytes and endothelial cells are situated near blood vessels and play a crucial role in the formation and stabilization of blood vessels. These cells are characterized by the expression of markers such as Platelet and Endothelial Cell Adhesion Molecule (PECAM), Lymphatic Vessel Endothelial Hyaluronan Receptor 1 (LYVE-1), and Regulator of G Protein Signaling 4 (RGS4) [44, 46].

Taken together, each of these stromal populations plays distinct roles in maintaining colon structure and function. However, how they are regulated and interact with each other or with the epithelial cells still needs to be better defined, particularly in pathological situations.

### Stroma-epithelium local signaling

As reported in the previous paragraph, normal fibroblasts play a vital role in producing ECM proteins, regulating the ISC compartment, and maintaining tissue homeostasis. These different functions are regulated by the expression and secretion of ISC niche signals that interact to control the fate of epithelial cells. Among the signaling pathways, it is essential to point out the WNT, BMP, EGF, and NOTCH, which play a fundamental role in maintaining the ISCs and epithelial homeostasis [2, 4, 45, 48]. Importantly, fibroblasts are dynamic cells that undergo “activation,” a process marked by the proliferation and secretion of growth factors and chemokines. Activated fibroblasts support epithelial regeneration and healing in response to damage or diseases [38, 48], such as intestinal epithelial repair *via* facultative ISCs stimulated by stromal factors like prostaglandin E2 (PGE2) [49]. Indeed, stromal PGE2, released after epithelial injury, recruits the facultative ISCs *via* the Prostaglandin E Receptor 4 (PTGER4) and activates the Yes-Associated Protein (YAP) pathway, promoting Leucine Rich Repeat Containing G Protein-Coupled Receptor 5 (LGR5)-positive active ISC regeneration [50, 51]. They can also help restore homeostasis through the secretion of RSPO3 [40] or by enhancing Insulin-Like Growth Factor (IGF) signaling by suppressing Insulin-Like Growth Factor Binding Protein 5 (IGFBP5) [52]. Moreover, fibroblast growth factors (FGFs), such as FGF2, are overexpressed following radiation-induced injury, enhancing ISC survival and preventing tissue hypoxia [53–55]. In addition to their action on epithelial cells, activated fibroblasts can also modulate the immune response by secreting molecules that recruit and activate monocytes and macrophages, thereby influencing the inflammatory status [38, 40]. For instance, in inflammatory bowel diseases (IBD), activated fibroblasts display epigenetic changes in the JAK/STAT pathway, which are responsible for chronic pro-inflammatory cytokine secretion (e.g., Interleukin 6 -IL-6-, Tumor Necrosis Factor alpha -TNFa-, Interleukin 1 beta -IL-1B-) [38, 56]. Of note, in IBD, the stromal sub-population that expresses anti-inflammatory cytokines (C-C Motif Chemokine Ligand 19 -CCL19-, CCL21, TNF Superfamily Member 14 -TNFSF14, IL-33, and PDPN) is nearly absent [40]. These unbalanced stromal cell populations in IBD surely account for the severity of the pathology and the difficulty of recovery. In these different settings, however, once the epithelium heals, activated fibroblasts may undergo apoptosis, return to their normal state, or become quiescent [40]. However, in cases of chronic damage, potentially due to epigenetic changes [40], fibroblasts may remain activated, contributing to disease progression, including cancer [40].

### CAFs in colon cancer initiation and progression

Intestinal fibroblasts have been shown to contribute to adenoma formation and colon cancer progression (Fig. 3B). These fibroblasts, collectively referred to as CAFs, represent a heterogeneous group of cells. Indeed, their precursors can be resident fibroblasts, epithelial cells, endothelial cells (through Epithelial-Mesenchymal Transition -EMT-), adipocytes, mesenchymal stem cells, pericytes, monocytes, mesothelial cells, hematopoietic stem cells, bone marrow cells, and smooth muscle cells [57]. Although their origins, markers, and functions may vary, most CAFs emerge in response to activation signals from cancer cells or the tumor microenvironment. Depending on the signals, CAFs can be categorized into (i) myofibroblastic CAFs (myCAFs), expressing high levels of ACTA2 [58], and (ii) inflammatory CAFs (iCAFs), expressing low levels of ACTA2 but high levels of cytokines and chemokines (IL-1, IL-6, IL-8, C-X-C Motif Chemokine Ligands -CXCLs-). TGFβ can induce myCAF transformation, while IL-1β promotes iCAF differentiation [38, 58, 59]. Single-cell transcriptomics reveals both normal-like fibroblasts and CAFs in CRC, but their classification, origin, function, and activation pathways remain unclear [59]. IL-1/JAK-STAT3 and TGFβ/SMAD2/3 behave as opposing pathways, inducing iCAF or myCAF formation, respectively [58]. Of note, the classification system for CRCs [60] identified four distinct molecular subtypes based on metabolic pathways, chromosomal instability, mutational status, or stromal cell invasion. One of these classes, known as consensus molecular subtype 4 (CMS4), accounts for 23% of CRCs and is characterized by a high concentration of stromal cells and activation of TGFβ signaling [60]. The transcriptomic signature associated with CMS4 shows overexpression of genes involved in ECM remodeling, EMT, and TGFβ activation, all processes closely related to fibroblasts in colonic pathophysiology [60]. These data suggest that CMS4 represents a CRC subtype where fibroblasts are the primary drivers of tumorigenesis [40]. In support of this assumption, *Tgfb*/*Bmp* gene alterations in mouse models induce intestinal tumorigenesis through the expansion of the ISC compartment and increased activity, accompanied by a concomitant blockage of cell differentiation [61, 62]. Accordingly, public CRC datasets have shown increased expression of GREM1, a BMP2 inhibitor, and a positive correlation between its high expression and a poor prognosis for CRC [63]. Additionally, mutations or loss of specific fibroblast-related genes, such as *Foxf2*, *Ikkβ, and Tpl2*, can enhance tumor formation, underscoring their role in tumor initiation [40, 64]. However, in addition to gene mutations, specific features of fibroblasts or their activation can favor tumor appearance. For instance, it has been shown that a subset of COX2-expressing fibroblasts can directly initiate adenomas [40] or that alterations in fibroblasts remodel the microenvironment, promoting adenoma formation through HGF/MET and WNT signaling [40].

Taken together, fibroblasts from healthy or pathological intestine play roles that go well beyond the classical role of cells that support the epithelial tissue. Activated fibroblasts and CAFs play an essential function in communicating with the other cell types composing the mucosa or the tumor. Their better characterization, new information on their activation mechanisms, and on the emergence of CAFs are surely crucial for clinical application.

## Stroma-epithelium TH-mediated signaling

This part of the review summarizes the current knowledge on TH-mediated epithelium-mesenchymal instructive signal exchanges. While the fine mechanisms of this signaling have been analyzed in detail during TH-dependent amphibian metamorphosis [20], comparatively little is known about mammalian gut physiology, and even less is known in pathological situations, including cancer. However, the process of fibroblast activation and its importance for tissue homeostasis or cell transformation in tumorigenesis is well established [39]. Given the role described for the THs and their receptors TRs in gut physiopathology, we provide in this section an overview of the current state of knowledge in this field.

### Intestinal physiology

#### The intestinal postnatal development during amphibian metamorphosis

Studies in amphibians have elucidated the cellular and molecular basis of TH/TR involvement in gut physiology during key developmental stages. Notably, larval-to-adult intestinal remodeling during metamorphosis is regulated by TH, serving as a model for mammalian intestinal postnatal maturation [65, 66].

Pre- and pro-metamorphic tadpole intestine (stage 54-57) [67] exhibit a simple tubular structure when plasma TH levels are low. At this stage, the lumen is lined with a single layer of larval epithelial cells, which are surrounded by connective tissue and a thin basement membrane. As plasma TH levels rise, significant changes occur: larval epithelial cells undergo apoptosis and are lost during the metamorphic climax [68, 69]. At early stages of metamorphosis, some larval epithelial cells that are precursors of ISCs (designated as preISCs) are induced by TH to dedifferentiate into ISCs [70]. These preISCs form clusters between connective tissue and surviving larval cells, rapidly enlarging and invaginating into the connective tissue to differentiate into adult epithelium as intestinal folds form by metamorphosis’ end [71]. Finally, the intestine develops a cell-renewal system along the trough-crest axis of intestinal folds, similar to the mammalian vertical epithelial axis [72, 73].

The cellular and molecular basis of intestinal remodeling in amphibians has been elucidated using in vivo and primary culture approaches. Specifically, THs are essential in mesenchymal cells to induce the formation of adult epithelium and ISCs [71, 74]. These findings demonstrate the key role of TH-dependent epithelial-connective tissue interactions in the development of an ISC niche in the metamorphosing intestine of amphibians [75, 76]. In this developmental program, TRα and TRβ receptors are crucial for fine-tuning the cell cycle, apoptosis, and ECM remodeling [76–79], particularly through the activation of metalloproteases [80]. In addition, various signaling pathways are regulated by TH in the developing intestine, including Sonic Hedgehog (SHH), BMP4, WNT, NOTCH, and Hippo, indicating evolutionary conservation in both amphibians and mammals. *Shh*, an early T3-responsive gene in the tadpole gut, is expressed in the epithelium and transiently increases during early metamorphic processes [81]. The connective tissue can respond to the epithelial-derived SHH signal through the activation of connective tissue-specific components of this signaling pathway, such as Patched, Smoothened, and GLIs [82]. Moreover, T3-dependent stimulation of the connective tissue induces *Bmp4*, another T3 target gene, which signals to the developing epithelium that expresses its receptor. This transient expression and stimulation of SHH and BMP signaling pathways in the epithelium and connective tissues temporally correlate with the adult epithelial cell differentiation [83] and with the suppression of cell proliferation in connective tissue [84]. Importantly, several TH-modulated genes involved in amphibian metamorphosis belong to the WNT pathway and are expressed in both the epithelium and the mesenchyme, thereby directly contributing to intestinal remodeling [85–87]. Indeed, the adult epithelium develops from a scattered population of larval epithelial cells that under the influence of increased TH levels and of both TRα and TRβ receptors, express *Ror2* and *Sfrp2* mRNAs. Thus, the induction by THs of noncanonical WNT5a/ROR2, canonical sFRP2/FZD, and hyaluronan/CD44 WNT signaling is required for larval epithelial cells to dedifferentiate and generate the adult-type epithelium [85–87]. In addition, in Xenopus intestine during Metamorphosis, YAP1 expression, an effector of the Hippo pathway crucial for mammalian intestinal homeostasis, is transiently upregulated by TH in adult ISCs and surrounding connective tissue cells, similar to CD44, suggesting YAP1 as a WNT target gene [88–90]. Interestingly, YAP1 is localized in the nuclei of some preISCs expressing ROR2 and adult ISCs, suggesting its role in the dedifferentiation of preISCs into adult ISCs and in their maintenance [91]. The NOTCH pathway, activated by ligand-receptor interactions between neighboring cells and upregulated by TH during metamorphosis [87, 92], involves key TH-responsive genes such as *Notch1*, *Hairy1*, Delta-like 1 (*Dll1*), and Jagged 1 (*Jag1*) [92, 93]. Interestingly, *Dll1* is expressed in the adult epithelium, and *Jag1* is present in both larval epithelium and stromal fibroblasts close to the ISCs. These distinct distributions indicate specific functions for the NOTCH components in the developing and adult intestinal epithelium as well as in the ISC niche [87, 92]. Taken together, TH-stimulated cell-cell interactions involving all these signaling pathways are fundamental for establishing the ISC niche required for the emergence of adult-type epithelial ISCs. Supporting this, a recent study highlighted TH-dependent signaling in tadpole telocytes, a fibroblast cell population described in the previous paragraph that expresses FOXL1 [94, 95]. Hasebe et al. demonstrated that TH indirectly upregulates *Foxl1* in fibroblasts *via* SHH signaling from the epithelium. Importantly, they showed that these signal exchanges between the epithelium and the FOXL1-expressing niche cells are needed for the emergence of ISCs in the tadpole intestine [96].

#### Intestinal development and homeostasis in the mouse

Driven by observations of TH-dependent postnatal development during metamorphosis in amphibians, several studies have investigated similarities in the developing mouse intestine. Functional studies on knockout animals during postnatal development at weaning indicate that the TRα1 nuclear receptor regulates the balance between cell proliferation and differentiation in intestinal crypt cells *via* WNT, NOTCH, BMP/TGFβ pathways [33, 97]. These functions of TRα1 are directly linked to its regulation of crypt ISC biology, as shown in vivo and in ex vivo organoid cultures [17]. The establishment of 3D organoid cultures has constituted a fundamental step in our understanding of the ISC biology. Sato et al. [98] described the formation of murine intestinal “mini-guts” from LGR5-expressing ISCs when maintained in a culture medium enriched with the stromal niche-derived factors essential for ISC viability and activity [98]. Indeed, FACS-sorted single LGR5-expressing cells can form 3D multi-budded organoids composed of all epithelial lineages when supplemented with EGF, RSPO1 (a WNT signaling amplifier), and Noggin (a BMP inhibitor) in an ECM-like gel [98]. These niche factors, together with vitamins and hormones, including T3, contained in N2 and B27 culture supplements, are necessary for ISC biology and effectively recapitulate the in vivo situation. In vivo, the ISCs are surrounded by a niche present at the base of the epithelium, which provides WNT proteins, RSPOs, EGF, and BMP inhibitors to maintain their identity and activity [1, 2]. The niche is composed of various cell types, including Paneth cells and stromal/mesenchymal cells (subepithelial myofibroblasts, non-muscle fibroblasts, telocytes, trophocytes, endothelial cells, and pericytes) [3, 5, 99]. It also comprises immune cells that interact with Paneth and M cells [5, 99], as well as the basal lamina, a layer formed by ECM proteins, which are essential niche components that participate in the complex signaling near the bottom of the crypts [100].

An action of THs and TRα1 was revealed in developing mouse intestine at weaning, coinciding with dietary changes and mucosal maturation. Mice with altered TRα1 expression (KO-models or TRα1-dominant negative mutants) exhibit reduced crypt/villus size [36, 101] and/or altered intestinal region lengths [18, 102]. This last aspect can be linked to the need to increase the absorptive and digestive capabilities. In addition, ISC activity is significantly affected by perturbed TRα1 expression or activity, resulting in altered differentiation properties [17]. These studies have focused primarily on analyses at the level of epithelial cells, with only marginal attention given to other cell types, such as fibroblasts, as is the case in amphibians. They had, however, underlined that TRα1 is also involved in smooth muscle contractility [103]. This aspect has become increasingly important over the last decade, following the discovery of *THRA* mutations in patients (see next paragraph). In addition, a recent study on a TRα1-mutated mouse strain (*Thra1*^*PV/+*^ mice, [104]) reported intestinal physiological impairments, including epithelial and smooth muscle defects, as well as reduced peristalsis [18]. The *Thra1*^*PV/+*^ mouse expresses a dominant-negative mutant, PV, which lacks T3 binding and transcriptional activity. The phenotypical analyses revealed defects in muscle fibers and interstitial cells of Cajal (ICC), disrupting electric activity stimulation and the intercellular transfer of molecules and signals. Indeed, *Thra1*^*PV/+*^ mice have reduced rectal smooth muscle contractility due to attenuated c-KIT signaling in ICC and significantly lower levels of key smooth muscle contractility regulators such as calmodulin, myosin light-chain kinase, and phosphorylated myosin light chain [18].

These different studies underline the key role of TH/TRα1 in epithelial maturation at weaning and in establishing adult ISCs. Recent advances have also highlighted the importance of epithelial/stromal interactions dependent on TH/TRα1, similar to those found in amphibians. However, we still lack information on the detailed mechanisms at play, particularly during the early phases of mammalian intestinal development involving smooth muscle differentiation. This is a critical point, given that the RTH-α syndrome in patients is characterized by decreased peristalsis leading to constipation and abdominal pain, as described below.

#### Intestinal human physiology and the RTH-α syndrome

The hormone T3 is crucial for human development and the proper functioning of many adult organs. Also, altered T3 levels are associated with human intestinal abnormalities and diseases, such as constipation and IBDs [105, 106]. Patients with TRα1 mutations have provided the first genetic evidence for the role of T3 signaling in the function of the human intestine [107–110]. RTH-α patients are characterized by nearly normal thyroid hormone and TSH levels, but they exhibit growth retardation, delayed bone development, anemia, and constipation [108–113]. These different abnormalities had been previously shown in mouse models carrying TRα1 mutations [101]. In particular, several studies have indicated multiple intestinal problems [97, 114, 115] in addition to decreased peristalsis [18, 103]. However, it remains unclear whether TRα1 mutations in RTH-α patients cause intestinal problems other than constipation. Importantly, the first TRα1 mutation was identified in a child who experienced acute constipation after weaning at seven months [110]. This result suggests that the TRα1 mutation may cause intestinal defects in human patients during intestinal maturation, particularly before and around birth, when T3 levels are high. This observation aligns with earlier studies that have demonstrated the critical role of T3 signaling in the development of the vertebrate adult intestine. We do not have additional information on inter-tissue communication defects in RTH-α patients at this stage. We can, however, speculate that genes involved in smooth muscle contraction, which are under the control of TRα1, are lost or less expressed, thereby causing constipation.

### Intestinal cancers: from mouse to human

In the previous sections, we have summarized the function of THs and TRs in intestinal physiology in amphibians, mice, and humans; here, we focus on intestinal tumors. Indeed, several studies have attempted to link the thyroid axis and the development of intestinal tumors. Nevertheless, contradictory results have been reported for both pro- and anti-tumoral effects of THs, particularly in epidemiological investigations [11, 116–120] (see the next section). However, model-based experimental data unequivocally demonstrate that elevated TH levels have a pro-tumorigenic effect [16, 121–123] and that mutations or altered expression of TRs may be linked to tumorigenesis [114, 124]. For instance, TRβ1 regulates cell proliferation in the liver [125], whereas it does not play a major role in the intestine [36]. Accordingly, in hepatic cancer cell lines, its elevated expression is strongly correlated with invasion capability [126]. In the colon, TRβ1 expression is reduced in cancer due to hypermethylation [127] and is associated with a more differentiated phenotype, whereas its loss is linked to malignant transformation [127, 128]. TRα1 is associated with pancreatic cell proliferation [129] and poor prognosis in breast tumors with mutated BRCA1 [130], while TRβ1 expression in the same study inversely correlates with favorable prognosis [130]. Our functional studies in mouse models highlight the role of TRα1 in intestinal development and homeostasis [33, 131] by regulating the WNT, NOTCH, and BMP/TGFβ pathways, as well as modulating cell division and cell cycle regulators [97, 132, 133]. The physiological relevance of these findings was validated by experimentally upregulating TRα1 expression in intestinal epithelial cells in vivo (*vil*-TRα1 mice), which resulted in the induction of pre-neoplastic lesions characterized by hyperproliferative and hyperplastic crypts [16]. In addition, the overexpression of TRα1 in a tumor-prone Apc-mutated model (*vil*-TRα1/Apc^+/1638N^ mice) accelerates the tumorigenic process and induces aggressive tumors [16]. Conversely, loss of TRα1 expression (TRα^0/0^/Apc^+/1638N^ mice) drastically reduces tumor incidence [123]. As in the normal intestine, key mechanisms include control of [133, 134] and functional interaction [115] with the WNT pathway. Recent findings in human CRCs show up-regulation of *THRA* gene expression in tumors correlating with WNT activity and associating with consensus molecular signature CRC subtypes [60, 123]. This comprehensive research provides a detailed understanding of TRα1’s role in intestinal development and cancer, with clear clinical implications.

**Fig. 4:**
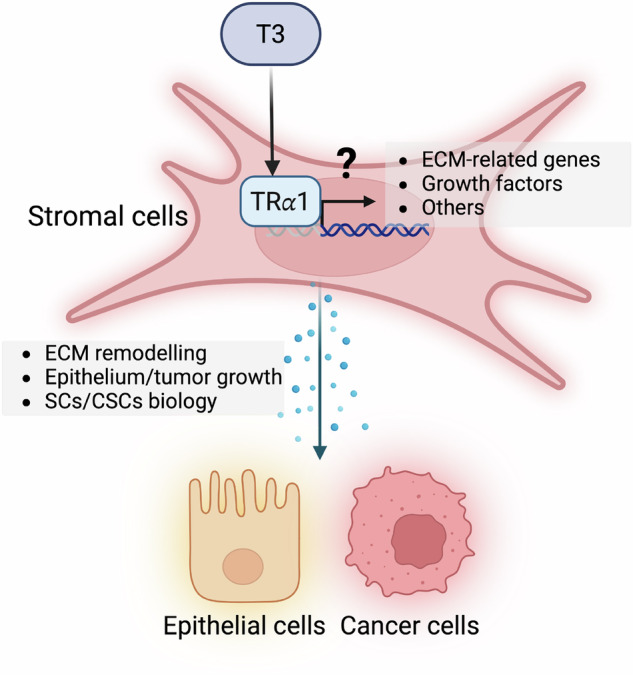
Proposed model of T3/TRα1-mediated epithelial/tumor-stromal interactions in normal colon and colon cancer. T3 *via* TRα1 can modulate the expression of ECM-related genes, growth factors, and eventually other genes involved in epithelial homeostasis and ISC biology. In tumors, where the expression of TRα1 is upregulated, these gene modulations may contribute to tumor growth and progression. Created in BioRender (Plateroti, M., 2025; https://BioRender.com/l3bpech).

Local TH availability is now recognized as a new and important area for the study of the TR-dependent intestinal tumors and CSC biology. Deiodinases (DIOs), the enzymes that metabolize the THs, have been examined in both normal physiology and cancer [135]. Tumor stromal cells from Apc-mutant mice show elevated expression of *Dio2*, a T3-activating enzyme. In this same setting, the loss of *Dio2* expression or chemically induced hypothyroidism blunts intestinal tumor growth and reduces angiogenesis [136]. This finding underlines the potential implications for the crosstalk between epithelium and connective tissue mediated by local or systemic TH levels. High expression of *Dio3*, a T3-inactivating enzyme, has been described in CRCs from patients [137]. *Dio3* loss-of-function in colon adenocarcinoma cell lines or primary tumor cells suggests T3’s pro-differentiation action through WNT downregulation and BMP pathway enhancement, leading to chemoresistance [137–139]. It is essential to note that *Dio3* has been reported in epithelial and cancer cells, while *Dio2* appears to be expressed by stromal cells, potentially including CAF cells. In this respect, we might speculate on an instructive crosstalk in which both deiodinase enzymes in different cell types play a key role in tumor biology. Also, these different reports underline the involvement and regulation by TH levels of signaling pathways (WNT, NOTCH, BMP/TGFβ) acting through different cell types by cell-cell interaction or by secretion of growth factors/signaling molecules. We may assume that, as in amphibians, this crosstalk plays an important role in tumor development and progression (see proposed model, Fig. 4).

Several studies have examined the impact of TH/TR on ISCs and CSCs. Macrophages and mesenchymal derivatives contribute to the ISC niche and the tumor microenvironment of CRCs. In these cells, THs regulate TREM2, a protein that acts in immune suppression [140], confirming previous findings on THs as immunomodulators, potentially reducing physiological responses against tumor cells [141]. TH/TRα1 influences intestinal tumor biology through interactions with the WNT/β-catenin and other signaling pathways [123, 142], possibly promoting tumor growth by maintaining the CSC niche. In support of this assumption, our recent studies have shown that T3 treatment or upregulation of TRα1 levels in human colon tumor spheroids induces a chemoresistant phenotype [143]. Intriguingly, this result is specific to FOLFIRI but not FOLFOX, two drug combinations routinely used to treat CRC patients [144]. Indeed, T3 treatment in the FOLFIRI setting interfered with cell death induced by the drugs, thereby maintaining the cells in a proliferative state. These effects are due to an action on CSCs and other cancer cell types, involving the stimulation of drug efflux transporters, finally resulting in decreased treatment efficacy [143]. Although informative and important, this study was performed in a simplified cancer model and did not include other cell types, such as stromal cells. To definitively demonstrate the impact of these results in CRC biology, similar experiments should be performed in a more complex and relevant colon cancer model in vivo or in co-cultures ex vivo.

The relationship between human cancer biology and TH levels is less clear. Due to their wide-ranging effects, THs may play a role in the initiation and progression of cancers by influencing cell proliferation and defense pathways. Beatson first noted a potential link between THs and cancer in 1896 by treating breast cancer patients with thyroid extract as a ‘lymphatic stimulant’ [145]. Since then, a large number of clinical, population-based, and experimental studies have attempted to clarify the possible effects of TH on the risk of cancer development as well as its influence on pre-existing tumors. However, 120 years later the first report by Beatson, this issue remains a matter of controversy [146]. Studies monitoring the thyroid axis in patients report associations between hyperthyroidism (low TSH and high THs) and cancers, including CRCs [120, 147, 148]. Most of those epidemiological studies monitored tumor incidence in patients treated with either TH-deprivation or TH-supplementation therapies, aiming to normalize TH levels [148–150]. However, these treatments do not mirror hypo- or hyperthyroidism and, paradoxically, show contrasting results, strongly contributing to the lack of consensus on this issue. Finally, multiple studies have shown that the hypothyroid condition induced by chemotherapy is positively correlated with higher survival rates, including tumor-free survival [146, 151]. Altogether, these findings clearly demonstrate the complexity of the field and underscore the need for additional, unbiased studies.

Some studies also suggest a noncanonical action of THs on tumor biology involving complex cell interactions, where THs modulate angiogenesis *via* the cell surface receptor integrin αvβ3 [152, 153]. This integrin is mainly found on tumor cells, proliferating endothelial cells, and tumor stroma-associated cells. TH/integrin αvβ3 signaling activates intracellular pathways linked to angiogenesis, mediated by classical pro-angiogenic molecules such as Vascular Endothelial Growth Factor (VEGF) [146]. Notably, the naturally occurring T4 analog tetrac (a T4 metabolite) blocks T4’s pro-angiogenic actions at the integrin receptor in CRC cell lines and is proposed as a promising clinical candidate [152]. These findings suggest a new CRC treatment route by blocking TH action, though further validation in more complex experimental settings is needed.

A significant advance in understanding the impact of TRα1 in human CRCs has been the demonstration of its up-regulation in cohorts of patients [123, 154]. In particular, TRα1 exhibits high heterogeneous expression in tumor cells, stroma, and immune cells [154]. More specifically, TRα1 up-regulation has been associated with the different CRC molecular subtypes (CMS), including those in which tumor/stroma/other cell interactions are important, such as CMS1, CMS2, and CMS4 [123]. Finally, and in accordance with previous studies, *DIO2* (which implies high intracellular T3) is strongly expressed in cohorts of CRCs, in particular in stroma cells [136]. It will be interesting to define the TRα1 expression levels in these same tumors and tissue types to link an eventual increased T3-dependent activity of TRα1 in specific tumor cells, CSC niche cells, and cells of the microenvironment.

## Conclusions and perspectives

In this review, we have highlighted the importance of TH-dependent signaling in intestinal physiopathology, with a specific focus on stromal cells as key target cells. Pioner’s works on TH-driven amphibian metamorphosis have described in great detail the cellular and molecular basis of this regulation in epithelial/stromal interactions. Interestingly, some key features can also be found in mammals, such as modulation of stromal-expressed WNT and/or BMP/TGFβ ligands [123, 133]. Importantly, alterations in mesenchymal derivatives can be observed in RTH-α patients, impairing proper colon functioning [108, 110]. Whether these malfunctions depend on altered epithelial-stromal communications is unknown and should be the focus of future investigations. Indeed, restoring correct crosstalk may represent a successful therapeutic intervention.

The *THRA* gene is frequently upregulated in CRC patients, in particular in molecular subtypes associated with enhanced WNT signaling and high metabolic characteristics [123, 154]. Importantly, TRα1 expression is upregulated in both tumor and stromal cells [154]; however, to date, we lack information on the eventual altered tumor/stromal interactions and their clinical relevance (Fig. 4). Also, there is a lack of knowledge of the relationship between normal stromal cells and the CAF regarding the TH/TRα1 signaling. We can speculate that altered TRα1 in both tumor and stromal cells may have an impact on CRCs, including decreased response to chemotherapies. A better definition of crosstalk and the factors involved is therefore of fundamental importance for more specific treatments of tumor cell targeting. Such new approaches could be included into the current therapies to increase their efficacy.
